# William James and the Sustainable Transformation of Values, with a Case Study for Rethinking the American Dream

**DOI:** 10.1007/s42087-020-00170-2

**Published:** 2021-03-11

**Authors:** Paul Croce

**Affiliations:** Stetson University, DeLand, FL USA

**Keywords:** American Dream, Covid-19, Economic Growth, Ideological Differences, William James, Practical Idealism, Psychology of Global Crises Conference, Social Transformation

## Abstract

This article addresses the call of the Psychology of Global Crises conference for linkage of academic work with social issues in three parts: First, examples from conference participants with their mix of bold calls for social transformation and realization of limits, a combination that generated few clear paths to achieving them. Second, presentation of Jamesian practical idealism with psychological insights for moving past impediments blocking implementation of ideals. And third, a case study of impacts from the most recent prominent crisis, the global pandemic of 2020, which threatens to exacerbate the many crises that had already been plaguing recent history. The tentacles of COVID’s impact into so many problems, starting with economic impacts from virus spread, present an opportunity to rethink the hope for constant economic growth, often expressed as the American Dream, an outlook that has driven so many of the problems surging toward crises. Jamesian awareness of the construction of ideological differences and encouragement of listening to those in disagreement provide not political solutions, but psychological preludes toward improvements in the face of crises.

The world is not designed in the way that the academic world is designed

Ian Parker (Parker, Crisis Talk)

Pluralistic idealism … a phil[osophy] I believe in

William James (2000), 8:57 

The American dream … has not been a dream of merely material plenty

James Truslow Adams (Adams, 1931)

The Psychology of Global Crises conference bridged the work of academics with the tasks of society. It was timely, conceived on the outset of the COVID-19 pandemic, and its online format brought the potential for public outreach. The organizers solicited contributions that would clarify “the concept of ‘crisis’” and asked, “what can the social sciences contribute,” with what “advice [in]… publicly communicated courses of action” toward just responses to many social crises? (Call for Contributions, 2020). These twin hopes, for a transformation of social values through both scholarly understanding and public action, often issued in tension between calls for bold transformation and for humility in the face of constraints. While these goals can seem contradictory, or even ones with potential to encourage retreat to one task or the other, they can actually reinforce each other, especially with the support of ideas from psychologist William James. He portrays boldness and humility in relation, with awareness of limitations keeping change agents motivated to persist through setbacks, and with the need for transformative action to push the hesitant beyond limited achievements.

This article addresses the call for linkage of academic work with social issues in three parts. First, a review of themes from conference participants with both bold calls and realization of limits, a combination that generated few clear paths to achieving them. Second, selections from the practical idealism of James about a range of psychological insights that can be enlisted to move past impediments blocking implementation of ideals through understanding of the sources of differences and encouragement of listening to those in disagreement. And third, a case study of impacts from the most recent prominent crisis, the global pandemic of 2020, which threatens to exacerbate the many crises that had already been plaguing recent history. The tentacles of COVID’s impact into so many problems, starting with economic impacts from virus spread, present an opportunity to rethink the hope for constant economic growth, often expressed as the American Dream, an outlook that has driven so many of the problems. Linking diverse crises and reframing the outlook that has been provoking them, shows application of these Jamesian ways to implement and sustain the energy from the conference and from similar impulses for realizing paths toward increased justice. This is not an attempt to solve the crises; such expectations for total solutions, from this view, are part of their problems; but these approaches can provide psychological preludes toward improvements in the face of crises.

## Thinking Big: The Raw Material of Conference Hopes

The goals expressed during the conference were aspirational. Participants supported ideals for how society should operate and deal with problems. Many remedies proposed seem readily within reach, but only in theory, because of political pressures and disagreements blocking them. The conference expressed idealism in identification of problems, especially inequitable class and race relations and unhealthy environmental practices. Presenters steadily pointed to the urgent need to pay attention to these issues, especially with COVID exacerbating them, or they proposed action steps not currently gaining much institutional support. They showed the idealism of noticing what individuals have little noticed or the idealism of promoting what structures have little worked for. This array of ideals provided the raw materials for action steps, as a sampling of examples from the conference readily shows.

Environment destruction and massive inequalities, despite their roots in centuries of violence and blunt profit seeking, Wade Pickren argued, have become normalized violence. But the shocks from COVID-19 might spur development of new norms for cooperation (Pickren, 2020). Similarly, Eva Vass and Alan Rayner hoped for new awareness of the “the iniquities … of globalised modern society,” with worldwide spread of the disease itself “a product of over-networking” (Vass and Rayner, 2020). Evelin Lindner likewise portrayed the pandemic as only the latest manifestation of a broader “dire predicament” generated by widespread “strategies of development that have shred our social fabric while simultaneously plundering our planet” (Lindner, 2020). Suorsa and Vadén endorsed these worries and identified their source in “energy blindness,” which makes many citizens, especially in the Global North, “oblivious and indifferent to the material basis of our existence.” However, the crisis may wake enough people from their “forgetfulness” that they will be ready to “act on current societal and environmental” problems (Suorsa and Vadén, 2020; and see Salminen and Vadén, 2015; and Suorsa, 2020).

Will pandemic-sparked attention to problems bring cultural change? Julia Libor saw potential during the economic slowdowns for “nature recovering partly” because of reduced traffic and less human enterprising in general. Perhaps, she suggested, these brief respites from routinized environmental damage can encourage “a new ‘normal’” with more appreciation of the natural world (Libor, 2020). Less optimistic, Thomas Teo portrayed the pandemic straining healthcare systems, with soaring death rates leading to “empathy selectiveness” and brutal decisions, not just in medicine, but more generally in “the division of humanity into humans and subhumans” especially when, even before the virus, “resources have been insufficient to meet everyone’s needs” (Teo, 2020). With overt idealism or with hopes delivered through warnings about ideals snuffed out, Libor and Teo reinforced the ambient conference calls to notice problems so long gaining little attention.

The idealistic hopes expressed at the conference also emerged in challenges to authoritative structures. Babette critiqued media assumptions “that the disease is caused by one thing, and a magic bullet will attack and solve it.” This “media epidemic” paralleling the health crisis keeps the world … focused” on the authority of experts even though this novel virus leaves “medical scientists … confused.” Despite this, alternative ways of understanding the virus, including inquiry into its complex ranges of factors, are “systematically bad mouthed” and “will not even be researched because … not profitable.” Maintaining support for the many benefits of scientific medicine, she points to the economic incentives that have “encouraged prescriptions” even when not needed, degrading the field over the last century. While this “dedication to domination [rather] than to health” has so often induced public compliance, she continues to hope that the sheer elusiveness and complexity of COVID-19 could lead to a humbler and healthier view of health experts (Babich, 2015 and 2020; also see Tomes, 2016).

In addition, the current health crisis could disrupt the structures of international relations with threats that have been largely ignored. Michelle Bentley depicted ways that this virus or similar diseases could be spread intentionally in biological warfare. This dire possibility serves as a reminder of vulnerabilities that need more attention, she argued, starting with more linkage of academic insights with public concerns (Bentley, 2020). Similarly, Kieran O’Doherty portrayed the pandemic challenging democratic politics, with fears of contagion reducing opportunities for citizens to gather and deliberate. Limited contact will amplify recent trends toward distrust of citizens espousing contrasting ideologies, even as democratic culture “requires trust to function” (O’Doherty, 2020). He hoped that some virtual public deliberations may enable continuation of this lifeblood of democracy with enough restoration of trust to support the social ideal of tackling serious problems.

The concluding session reviewed the high hopes expressed during the conference, including the capacity for the pandemic to shed light on many problems too often ignored and the potential for the crisis to spur structural reform. Kenneth Gergen heard the big ideas presented over the previous few days but insisted on attention to the constraints on implementing them. He praised this “landmark” gathering but threw down a challenge to participants and audiences: “do we become part of the babble in public or do we have … a new agenda … to offer [for] moving the world forward, … and what does that mean”? (Gergen, Crisis Talk). This was a challenge that went to the core of the conference mission for bridging the scholarly and public worlds and for mediating the messages about both boldness and humility. How implement high ideals while addressing contrasting impulses and keeping each from hampering the other? Gergen’s chastening charge served as a reminder that ideals for directing more attention to injustices and for their reform are necessary steps, but in themselves insufficient for social change. The next crucial ingredients are an understanding of the ideological diversity blocking these hopes, insights on the construction of these divergent perspectives, and practical plans for dealing with their contrasts.

## Jamesian Impulses for Turning Ideals into Action

The psychologist William James (1842–1910), who was himself one of the founders of the field but at the same time an engaged public intellectual, provides insights for building bridges between scholarship and public life, and for integrating hopes for social transformation with realization of limits on these ideals. He first learned to mediate contrasts in his own youth when he was torn between his father’s religious thought and the methods of his scientific education, and troubled by still more tensions about his personal direction and philosophy of life. After prolonged struggles, he turned his ambivalence about the contrasting choices into a decisive ambivalence about the merits and potential usefulness on each side (Croce, 2018, 26, 182, 245, and 270). And he turned this change of attitude into a research program for assessing how the differences emerge in different people and for different purposes. He called this “the psychology of philosophizing,” and his central point was that each worldview has roots in the sentiments that allow each person or group to feel most “at home” in particular ways of thinking. In his “Sentiment of Rationality,” he proposed that the sentiments beneath each commitment, even when developed into rational discourse, help explain not only disagreement, but also the sense of priority and motivation for each view held (James, 1978, 33). In his psychology, James portrays mental attention driving these commitments by amplifying or curtailing parts of experience. Experience presents a super abundance of facts, ideas, and points of view, a “blooming buzzing confusion” more than anyone can take in (James, 1981, 462). Traditions of one’s upbringing, aesthetic attractions, and emotional appeals encourage selective attention to those experiences of particular interest.

The flip side of attention’s focus on parts of experience is the ignoring of other parts “not needed by the intellect” for specific purposes; they are therefore “shunted off” (James, 1981, 431). For example, when people disagree or when the public does not heed the insights of scholarly research, their focus may be shaped, as James put it, by “more nutritious objects of attention” than what one strand of research can provide (1981, 518). James’s perspective suggests a way to move beyond dismissal or anger toward those who disagree. Outside the conference, historian Tim Lacy, himself using James, argues that these hostilities are a signal and result from “failures of discourse” (Lacy, 252 and 265). At the conference, John Clark and Brendan Jacobs lent support to this outlook in evaluation of the impact of the pandemic on schools and in public interaction. Recognizing the prevalence of sharply different ideological points of view, they portrayed this or any “crisis as a signal of a conflict unresolved,” with “opposing views … complementary,” even when “on their face they look like opposition.” Borrowing from “Aristotle’s law of the excluded middle,” they suggested that “mutually exclusive” values in contrast can be understood as “actually mutually inclusive.” With this thinking, they expressed hope for encouragement of steps “to get beyond separation” of contrasting ideas and people (Clark and Jacobs, 2020). With this thinking, as with James, questions would be, What is in fact supporting those contrasting outlooks, and how can they be expressed with acknowledgement of the contrasts, even without any compromise of one’s own views or values? For turning the conference ideals toward practical actions, Jamesian ideas offer suggestions about the benefits of paying frank attention to the sources of contrasting ideologies, including consideration of both how opponents think and the sentiments of one’s own rationalities. This can illuminate the ways each ideology appeals or does not, and even ways that the sentiments of others, harboring rational treads in domains previously ignored, may be potentially helpful to one’s own cause.

For encouraging the implementation of ideals, James takes a layered approach to contrasting ideas. When introducing pragmatism in his 1898 lecture, “Philosophical Conceptions and Practical Results,” James used a metaphor derived from his fondness for hiking with the whole forest equated with the full range of experience available in everyday life and for all interpreters. Particular theories or beliefs serve as blazes and trails through the woods. These are enormously important because with them, “we can now use the forest…. It is no longer a place merely to get lost in.” Yet, despite their great usefulness, these trails are not themselves, he points out, the whole “trackless forest of human experience.” He reminds his audience that even the most brilliant mental trails “leave… unexpressed almost everything in the forest,” which as a whole remains out of human reach. With this metaphor, James frames his pragmatic philosophy with two levels of truth, happening at the same time and each valuable. The whole truth, all that could be known, remains mysterious, while particular truths, represented by the paths, explain parts of that whole; the paths “circle toward the … direction” of the total truth. He took pride in the often-impressive paths of human achievement but maintained humility about the “ever not quite” of each of these compared to the whole (James, 1975, 258). This model shows simultaneous awareness of both the value of each human achievement, including proposed ideals, and their limitations. This awareness of human limitation is not a reason to be crestfallen but to consider the different dimensions in relation to each other. Each proposal for greater justice with focus on its ideological trail, can gain from realization of its relation to the whole, including humility about its limits and strength from connection to that broader perspective. James heartily endorsed moral uplift from the world’s many ills, even while recognizing the partiality of many trails of human attention and experience. This combination prompted his earnest will to achieve ideals and his openness to corrections about the best methods for addressing those ills and about challenges from the views of others.

James takes up this range of human perspectives in his theory of pluralism. He applauds commitment to ideals, even as he warns about treating any set of ideals as “fixities,” because they serve as only “snap-shots” compared to the “continuous … flux of life” (James, 1977, 106, 105, and 109). Keeping aware of the full plenum of truth, the whole forest beyond any one trail, is not only an encouragement for humility. In addition, this awareness can provide “living sympathy” with others who each “interpret … in such diverse ways,” both allies with different methods for achieving common goals and antagonists who oppose those goals because selecting different parts of experiences (James, 1977, 117). This awareness can also take the burden away from any one person—or point of view—to be held accountable for solving the whole. His outlook suggests dispersed responsibilities. And it can reframe the conflicts between different ideologies or between average citizen and academics by asking each to apply their potential expertise to their specific parts of experience. Rather than standing in firm opposition, each point of view can be put to work addressing particular problems instead of expending energy only on antagonism. With each problem enormously large, each with its own “trackless” complexities, these insights can help to keep both individuals and groups commited, with each playing a part of a larger whole. In addition, this approach can spur tactical awareness of thoughts in opposition for moments of competition and persuasion, and it can avoid the despairing feeling that any one contribution is too small to be significant. The mediation of parts and whole in Jamesian outlooks can enlist many diverse citizens in the tasks of social uplift. “Each attitude being a syllable in human nature’s total message,” James proposed, “it takes the whole of [them] to spell the meaning out completely” (James, 1985, 384).

Throughout his works, James not only mediated contrasting views; he also expressed further support for his goal of turning conflicts into opportunities, by understanding the sources, operation, interaction, mutual limitations, and need for careful scrutiny of different outlooks. And his successors in psychology and related fields have honed insightful and constructive guideposts, in the spirit of James’s insights. Like James, they are theorists about action, with theories that can help support action.

At the conference, Ruthellen Josselson concluded with lessons from her experience in psychotherapy, especially couples therapy, to shed light on cultural conflicts. When individual troubles weigh on relationships, they often spur conflict. But an understanding of the sources of the conflict in the individual troubles can help each side grow—with problems even becoming opportunities supporting the development of both the individuals and the relationship. This is a helpful context for understanding how members of groups come to disagreement, and this supported her proposal to encourage attention to the views of people we decry, even if we think, as Michelle Fine said pointedly in the same concluding session, “their software needs rearranging” (Fine, Crisis Talk). For this, Josselson suggested, it helps “to be humble” because this posture can both generate understanding and even increase power, since inviting insights foreign to our own can help each of us “find what we don’t know” (Josselson, Crisis Talk). While these approaches are sometimes greeted as calls for consensus or invitations to accept or justify outrageous views, these criticisms miss the point about communicating across differences; listening does not mean accepting. James’s ideas about the sentiments in other people’s rationalities support Josselson’s readiness to learn about the sources of conflict. Like James, she is providing portraits of the construction of contrasting outlooks, each based on different premises and each promoting selection of different facts. Taken together, Josselson and James show kinship to other contributions from the conference pointing to kindred ways that disagreements can serve as signals for discrete but complementary action steps toward social ideals. And these ideas support agonistic interpretations of democracy, with even sharp differences, provided they are treated with respect, actually improving political health. This represents a step beyond tolerance; as with Josselson’s clients, identifying cultural differences can be sources of clarity and growth. Philosopher William Connolly calls “agonistic respect … a cardinal virtue of deep pluralism” and therefore crucial for the health of diverse democdracies (Connolly, 125).

At the conference, Molly Andrews heard possibilities like these from Josselson, James, and Connolly and praised them for their hopefulness but pointed out that this stance is not synonymous with optimism. This suggests the need for sustaining focus and purpose even through difficulties, while maintaining effort to realize goals. Environmentalist David Orr expresses this idea vividly, “hope is a verb with its sleeves rolled up.” Rather than focusing on the difficulties, he urges “redirect[ing] energy … in a positive way, … by DOING stuff” (Orr, 2010). Andrews tempers her own boldness with humility in urging both avid “use [of] our expertise,” while also “realiz[ing] that we do not know everything.” Especially with this combination of the bold potential of intellectual prowess and humility about limits even among the experts, she suggests choosing direction carefully because “we are at an acute turning point.” In particular, she urges assessment to discover “of what kind” that turning point is; in other words, plan ahead (Andrews, Crisis Talk). James put the name “meliorism” on the persistence toward betterment that Andrews advocates without waiting on optimism, in his similar pursuit of idealistic ends, even as he did so through different means. His unblinking awareness of the often-elusive whole of experience expressed the spirit of Andrews’s humility, even as he insisted that many answers do not emerge in the abstract, with prior planning, but through the actions themselves. He explained this as a future-oriented feature of evolution in general, with the emergence of new species not by fulfillment of prior purpose and also not in purposeless uncertainty, but by addressing the immediate purposes of each particular setting. In the same way, he pointed out that actions taken to address specific problems, even when facing uncertainty about their efficacy, can serve as living laboratories for both motivation to keep on trying and the constant correction he had learned from the scientific method. This is counsel for practical action not just despite uncertainties, but because of them. He portrays purposes emerging not before the actions or in abstract planning but remaining inchoate until facing actual problems themselves, then taking shape in response to those particular problems. In her call for actions with purpose, Andrews shares his humility about any total answers, even as he applies that humility in a practical way by urging attention to purposes that emerge from the actions taken rather than before them.

Mark Freeman spoke to a paradox in academics’ relation with society. Reflecting frequent public impatience with the work of scholarship, he admitted that it “often feels abstract and self-enclosed,” yet for the support of social ideals, he added that he does “not want to give up on what I do” (Freeman, Crisis Talk). He also expressed his resolve that action means “not [staying] content just to write better articles” and his frank hope to “focus … on people different from me,” outside of academia, even as he remained unclear about “what … it mean[s] to get in the game” of public engagement (Freeman, Question and Crisis Talk). In his own session, Freeman identified one important role for the learned class: Because only “some have the luxury of hope because hope is distributed unevenly,” both by class and race, academics can serve those out of power by evaluating the structures of power and giving voice to the voiceless. But he felt a barrier emerging on this path when noticing that many of those out of power support policies of injustice; he pointed directly to supporters of the current presidential administration. From Freeman’s perspective, President Donald Trump’s ideas and actions “are monumentally absurd,” even as he recognized that they appear so only to “those who believe [they are] monumentally absurd” (Freeman, The [Al]lure of Narrative). This shows a tacit recognition of James’s proposition about “a certain blindness in human beings,” which inhibits understanding of fellow human beings who live and think with very different assumptions; those differences can, and in polarized cultural climates often do, prompt hostility (James, 1983). Jamesian insights about the sources of respective assumptions, mutually attributed to “blindness,” but mutually emerging from attending to different selections of experience, can serve as steps toward understanding and even learning across differences.

Freeman’s own puzzlement over profoundly different worldviews suggested his own story about debates with his daughters. When he would go into “contempt mode,” with hostility for people with outrageous views, they would “rebuke” him, urging that he “show respect for people who are different.” Freeman fully admires the tolerance and capacity for fellow feeling that his daughters display, reflecting his own affinity for Jamesian recognition of the different “sentiment[s] of rationality” that different people readily feel “at home” in and use to oppose each other. But his daughters have not yet fully persuaded him because his outrage for outrageous views is based on very real problems in race relations and the environment. In the face of these facts, he asks, “how is it possible… not [to] care about reality,” to “resist … knowing”? Here, he speaks with the conference-wide boldness about commitment to ideals. But his own consideration of ideas in relation, as he writes in *The Priority of the Other*, especially with his view of different people as the “mystical other,” suggests another path (Freeman, 2014, 146). In the book, rather than seizing on (his own) correct solutions, he calmly considers ideas as they appear to others. He calls this his “hermeneutics of hope,” with compassion toward those others, those “‘thems’ we might despise,” even as he himself honestly struggles to maintain this posture himself (Freeman, Crisis Talk). James would both endorse this compassion across the ideological divides and provide a way to address Freeman’s outrage, which is so representative of intense stances across those divides. The earlier psychologist was ready to apply the “ever not quite” of his metaphorical hike through the trackless forest of human experience not just to the shortcomings of others, but also to himself. From that perspective, even those with outrageous views are just fellow fallible humans; and somethings about those views or the way they appeal might even provide clues about one’s own shortcomings.

In addition, Freeman’s endorsement of the power of art offers further opportunity to mix boldness with humility because of the ability for works of art to address feelings, which can cut through the crust of misunderstanding, distrust, and limited empathy. James’s awareness of the ways that ideological positions, even when expressed rationally, have roots in sentiments of feelings, aesthetic attractions, traditional affiliations, and personal commitments, fully supports Freeman’s turn to artistic expression. And this path offers a response to Freeman’s own dilemma. Freeman can continue to issue rational critiques with outrage against positions he finds absurd, but that is not the only mental tool available to him or to any of us. The Jamesian perspective is that touching people emotionally triangulates between contrasting ideologies, bringing potential shifts where exclusively rational discourse might have little impact. Reasonable debate, when soundly argued and supported by facts, persuades many, but can become even more effective when accompanied by a moving work of art or story, or an appeal to prior commitments.

The combination of robust energy for taking action and endorsement of uncertainty and limitations appeared throughout the conference. Clint Burnham presented a way to use each, with an appreciation of these in social levels paralleling James’s levels for understanding truth. Social responses to the pandemic, with work from home and homey intrusions into public space, Burnham noticed, show both an amplification of a tendency already happening in the digital age, and a modern version of a medieval theory about the relation of public and private domains (Burnham, Crisis Talk). He referred to the theory of Ernst Kantorowicz, who proposed that the king had “two bodies,” the public body with governing power and formal presentation in pristine form, and the physical body full of vulnerabilities and subject to social challenges (Kantorowicz, 2016). This distinction also has potential for negotiating ideological differences and for providing suggestions about steering through crises in general, because similarly, each citizen has “two bodies.” Each can be a supporter of an ideological group and is at the same time a citizen of the whole country, and these can lead to citizen as advocate of bold transformation based on a particular ideology or as one person among others humbly ready to acknowledge limitations from need to work with the diversity of the whole population. This shows a parallel to James on truth, with partial truths appearing in bold ideological commitments, while the broader truth of citizenship serves as the elusive whole toward which each ideology strives and can potentially contribute to. Burnham’s ideas also point toward a way to mediate these contrasts by again considering a theory emerging from one setting for application more broadly.

The social theorist and activist W. E. B. Du Bois, who had studied with James, developed a theory of “twoness.” Du Bois astutely observed that, because of prejudice, African Americans need to circulate on two levels, one from within their own subculture and also as citizens of (mainstream white) American society. Twoness emerging from social tragedy has also provided opportunities for special insight. This hard-won awareness of simultaneous levels suggests a way to challenge contemporary advocates of transformative social ideals to combine critiques of policies hostile to their reforms with detection of the values motivating their antagonists. Listening through and despite disagreements will dampen the thrills of cultural warfare while keeping eyes open to the values commitments of ideological opponents. This approach to twoness can support calls for social transformation while acknowledging the challenges of ideological diversity. The first level involves the commitment of James’s “will to believe” in support of the “faithful fighters of this hour,” while the second taps his psychological awareness that each person is a “creature of superb cognitive endowments” with capacity for great “subjective spontaneity” leading to the “luxuriant foliage” of so many ideological commitments—for good or ill (James, 1979, 56; 1978, 12 and 14). A more thorough social transformation awaits the advocates for social transformation ready to engage with those variations. Without tapping the twoness, the very act of achieving important ideals, with victories gained by ignoring or defeating those in opposition, would dissolve the democratic need to maintain respect for all citizens even when in disagreement and would lessen the ability to implement ideals.

## A Case Study for Practical Application of Ideals: Rethinking the American Dream

For the conference charge to take up the task of connecting depths of learning to depths of problems, many participants proposed suggestions and urged action steps, especially about large social and environmental problems, but suggestions generally remained conference ideals. Jamesian theories provide a range of insights supporting efforts to implement these ideals, including raising awareness about how ideological commitments emerge, how they can avoid their own blind spots, how they can foster respect through disagreements, and even how those in disagreement can be enlisted to take on parts of the problems, but Jamesian theories remain ideas about action. How can these ideals and ideas turn into action? To support this task, Jamesian approaches to ideological diversity suggest consideration of the ideological factors that have served as roadblocks to implementation of these compelling hopes.

In particular, I propose that the widespread and generally undebated commitment to constant economic growth, especially the appetite for constantly more consumer goods, has served as a deep driver to the injustices, especially environmental degradation and hierarchies by class and race, identified by the conference participants and many other commentators. And a major reason this revered commitment has remained assumed without widespread debate is that it has been understood as part of another cultural ideal, the American Dream. In the USA and in transnational views of the USA, the democratic hopefulness about release from oppression has been overwhelmed by materialist views of the American Dream with avid consumption of goods. The call here for renewal of the original democratic meaning of the American Dream offers a specific action arena, a place where a theory, the persistent cultural ideal for constant growth, a theory straying from its own democratic roots, has shaped actions enormously and blocked potential for even noticing problems, making transformation both politically difficult and culturally unimaginable to many citizens. Attention to those competing ideals can serve as a first step toward implementing the ideals that participants of the Global Crises conference, Jamesian commentators, and many others have been advocating, but so often with limited impact. In addition, challenges to the tradition of constant growth, because it is so widely dispersed and assumed, can be taken up by average citizens and scholars alike—an avid hope of many change agents—at numerous points in personal and social life. The very breadth of reach of the American Dream means there are many opportunities for transformation, both individual and structural.

The American Dream circulated before the phrase was coined in 1931 by historian James Truslow Adams (1931). “Avant la lettre,” in the French phrase, Americans and their admirers around the world believed that in this land without the confining impediments of traditional hierarchies, the poor and oppressed could rise in station based on their own abilities and hard work. Adams expressed confidence, even at the outset of the Great Depression, that the traditional American social dynamics for improving one’s station in life applied to “any and every class.” But these opportunities for social mobility mostly went to white male Protestants. This hierarchy displays that the reputation about individual merit at the heart of pride about the American Dream has been shot through with differential structural advantages. Women, displaced Natives, and enslaved African Americans lived with the cruelest opposite of the American Dream. Their abilities brought sneers, and their hard work rarely brought rewards. In 1964, Malcolm X made this point sharply about the experience of most non-whites: “I don’t see any American Dream; I see an American nightmare” (Blackpast, 2010).

In 2020, massive surges in unemployment brought by COVID-19, with a 3000% increase in US jobless claims in the first month of the pandemic, have meant that the disease has expanded the number of American workers, beyond those put down by prejudice, not able to reap the fruits of the American Dream (Kelly, 2020). The shock of more citizens blocked from material acquisition can serve as a reminder of the deeper roots of what the phrase and its promise of opportunities have meant. Even with all of its burdens and tragedies, the COVID pandemic provides an opportunity to reclaim the depths of democratic promise embedded in the American Dream beyond constant material gain.

Environmentalists call recent times the Anthropocene Era, when the human species has brought enormous impacts on planet earth with climate change, loss of species, and wholesale transformation of landscapes, but also with enormous cultural and social achievements (J. McNeill and Engelke, 2016). Those impacts are real, but these times are also a small chapter of what could be called the Microcene, a much longer era of earth dominance by microscopic viruses and bacteria. Compared to the successes of the human primate, these creatures are more adaptable, and as William McNeill recounts, they have had significant control over human history (W. McNeill, 1998). Human development has even enhanced the strength of viruses and unhealthy bacteria effectively creating a veritable Microcene Dream for microbes with limited opportunities. Countless microbes had long remained confined to local settings, but as human habitations have spread, greater contact with wildlife has provided these pathogens with opportunities to migrate out of their old locales (Osaka, 2020). The resulting increased zoonoses, diseases transmittable from animals to humans, make full use of modern human practices. Globalization provides highways of transmission, and cities, crowded markets, and refugee camps increase potentials for contagion (UN Environmental Programme, 2020). The diseases are natural, even as human practices amplify their spread and virulence, providing examples of what Ted Steinberg calls the “unnatural” components of natural disasters (Steinberg, 2006).

This pandemic, Farhad Manjoo points out, has alerted Americans that “our society might be far more brittle than we had once imagined” (Manjoo, 2020). Other colossal problems lurk, ranging from skyrocketing healthcare costs, eroding infrastructure, and racial tensions, to potentials for nuclear war and terrorist strikes while sea levels rise, fisheries and arable land erode, and toxins leach into waterways, to name only a few of the potential disasters, human-made and natural. Any one of these structural disruptions could block individuals from striding toward their slice of the American Dream. This indeed has been the impact of many recent disasters, but only regionally, with hurricanes on the Gulf Coast and Eastern Seaboard, floods along the Mississippi River, and fires in the western states. The only strangeness of these strange times may be that it has taken so long for one of these massive disruptions to erupt on a national scale.

These grim possibilities highlight that the American Dream and its success stories have been skating on assumptions about a generally healthy, undisrupted, and peaceful society, with the most privileged able to reap riches from these beneficial conditions, and those able to board the escalator of social mobility ready to join the bounty. When these Americans, or those aspiring to their conditions, have faced disasters before, ranging from other epidemics, hurricanes, and earthquakes to depressions and wars, they could readily notice that most of them have been localized, temporary, managed, diverted, or offshore, as with almost every American war.

This history has encouraged a sense that such problems are exceptions to the regular smooth operations of society, not factors to consider seriously compared with the abundance of opportunities. Even the tens of millions of deaths worldwide from the 1918–1919 Spanish Flu, until recently a largely forgotten pandemic, did not dent this powerful American self-understanding (Crosby, 2012). For hundreds of years, looking past disasters has been the norm for American majorities. Americans can weather the setbacks, according to these avid supporters of the American Dream, while the dream has endured because better opportunities always seemed just around the next corner.

The American Dream, with its assumptions about resources readily available for human enterprises, has shown little reckoning with the social or environmental impacts of those human dreams. In fact, this traditional way of using resources displays what James called a “wishing-cap” approach to life: He referred to the “apps” available in the early twentieth century: “We want water and we turn a faucet. We want a kodak-picture and we press a button. We want information and we telephone.” Fast forward, fancier apps, but the point is the same: the technology is remarkable; then, rather than having to think about the sources or implications of our wishes, “we hardly need to do more than the wishing—the world is rationally organized to do the rest” (James, 1975, 139). The system is well suited to addressing the material parts of the American Dream but is not well built for supporting equitable social relations or sustaining the environmental services that contribute to material prosperity.

Living with comforts, enjoyment, and creativity does require resources, but these do not need to be on very large scales. Thomas Jefferson endorsed the virtue of “a middling level of development,” with freedom both from the pain of want and from the burdens of managing extensive holdings (Katz, 2003, 8). For the next chapter of human history, the challenge will be: How to maintain the continued flow of the American Dream’s democratic appeal with fewer of its material problems. How to cool the constant pressure for more material things, which has been tempting fate by inviting material disasters, but continue to provide broad opportunities for sufficient material resources and for enrichments of more parts of life beyond the stuff? The system of constant economic growth really has been wonderful for many people, even as it also has produced enormous side effects on those without opportunities and on the landscape. Attention only to the wonders turns a blind eye to the other effects of constant material growth, including the very problems highlighted by participants at the Crisis Conference.

Most of the side effects of the association of the American Dream with constant growth are problems of scale, and they show the intimate and challenging interrelations of individual and structural factors in potentials for social change. Each individual act of pollution, while not good, is not world destroying. But on mass scales, they can become overwhelming disasters, much like viruses expanding beyond local settings. Each bottle not recycled, each errand run by car rather than on foot, each cigarette butt tossed on the ground has close to zero environmental impact; in statistics, they are treated as effective zeros. But when ignored by individuals and by systems with no incentives for healthier actions, the collective mass creates destruction. Yet changes by individuals and structures, even when small at first, can add up. As James says, there is “legitimacy [in]… some” (James, 1977, 41). That is true many times over in mass culture. Plus, taking one step at a time can not only avoid some of the difficulties of change, but also it can recruit the hesitant who feel humbled about their own limited power or overwhelmed by powerful structures supporting the problems. While many problems require structural transformation, individual actions offer support to those broader changes as building blocks and cumulative prods toward more thoroughgoing reckoning with problems. Enough structural and individual changes can lead to tipping points.

For example, individually, average consumers can try to live within their means buying the durable rather than the disposable; business owners can strive to maintain healthy returns without adding overly risky ventures; homeowners can buy houses with ample space rather than with areas mostly unused (and hard to maintain); presents can be for experiences rather than for things; healthy people can engage in preventive care, and when getting sick, they can try the least-invasive treatments first, holding off on major medical interventions, in the spirit of Babich, until needed (Babich, 2015 and 2020). Structurally, manufacturers can design products with reused materials or at least with materials that can actually be recycled or reused; incentives can encourage advertisements for healthy cultural attitudes just as we currently have so many ads with attitude about otherwise mundane products; institutions can reduce use of fertilizers, insecticides, and leaf-blowers on their lawns, while mowing less, with some increased tolerance for the aesthetics of the wild. These examples of what Duane Elgin calls “a way of life that is outwardly simple [but] inwardly rich,” are not portraits of life in hardship, and they do not offer uniform formulas (Elgin, 1993). But these samples suggest the potential for more thorough relishing of the material things we do have before seeking more things, just as Lindner and Pickren hoped.

These suggestions also serve as reminders that the effort of trying to get more things actually takes a lot of time, which is ultimately the most valuable resource in life, especially when the more things gained might actually lead to more annoyances or troubles. With more lesserism, there is no need to adopt the minimalism, insurgent or fashionable, that Spencer Kornhaber has scouted. Certainly, calls for less consumption is not applicable for those living on a minimum already, but use of fewer things can help those of middling station “think critically about what’s necessary and what’s not,” encourage those with more to use their abundance to help the desperate, and support individual and structural efforts to enable more to gain sustainable self-sufficiency (Kornhaber, 2020).

The sooner more people can rally to finding riches beyond the material, the sooner we can figure out the healthier paths. Listening to those with different values and across different sentiments can encourage paths toward less squeezing for more material gain and more ability to achieve more justice. That would mean more attention to the less-material rewards of the American Dream with its democratic hopes for more people to gain more uplift and its potential to enable more personal development, healthier social relations, and more creativity unhitched from the need for lots more things. Without these changes, the boulder of widespread assumptions about the value of constant growth will make many other calls for change stillborn, including the calls for transformation at the conference. And without the personal integrity of personal commitment, calls for structural change will invite widespread ridicule, feeding the narratives of those beating drums for the status quo. Just as the American Dream of constant growth has been a driver of so many of the injustices aimed at by so many hopeful ideals, so a revision of that dream mentality can support those moral causes and contribute to the potential for groundswells toward structural transformation.

James’s portrait of human psychology presents a range of supports for promoting a sustainable approach to such changes. The social habits for steady increase in material goods are strong, but the changes with COVID and other lurking crises may encourage rethinking of those traditions, with tangible preaching that may be more persuasive than many sound arguments so often overlooked. And for such changes, we can rely, as James pointed out, on humanity’s “extraordinary degree of plasticity” (James, 1981, 110). COVID-19 has been a disaster and created or amplified many crises; it also presents an opportunity to continue dreaming with less destruction and with less vulnerability to current or future crises. An American Dream reclaiming its democratic roots and growing into structural reform can gain support both from academic ideals and from countless (limited or humble) individual actions. That democratic dream of expanding opportunities can reduce the severity of crises in human relations and in the natural world that its more aggrandizing cousin, with appetite for constant growth, has been promoting for years. Rethinking the American Dream presents a central opportunity for using Jamesian psychology about the formation, interaction, and potential evolution of ideologies to encourage realization of transformative ideals including those expressed so forcefully at the Psychology of Global Crises conference.
